# What Is Nuclear Factor Kappa B (NF-κB) Doing in and to the Mitochondrion?

**DOI:** 10.3389/fcell.2019.00154

**Published:** 2019-08-07

**Authors:** Benedict C. Albensi

**Affiliations:** ^1^Division of Neurodegenerative Disorders, St. Boniface Hospital Research, Winnipeg, MB, Canada; ^2^Department of Pharmacology and Therapeutics, Max Rady College of Medicine, University of Manitoba, Winnipeg, MB, Canada

**Keywords:** inflammation, immunity, mitochondria, cancer, Alzheimer’s, nervous system

## Abstract

A large body of literature supports the idea that nuclear factor kappa B (NF-κB) signaling contributes to not only immunity, but also inflammation, cancer, and nervous system function. However, studies on NF-κB activity in mitochondrial function are much more limited and scattered throughout the literature. For example, in 2001 it was first published that NF-κB subunits were found *in* the mitochondria, including not only IkBα and NF-κB p65 subunits, but also NF-κB pathway proteins such as IKKα, IKKβ, and IKKγ, but not much follow-up work has been done to date. Upon further thought the lack of studies on NF-κB activity in mitochondrial function is surprising given the importance and the evolutionary history of both NF-κB and the mitochondrion. Both are ancient in their appearance in our biological record where both contribute substantially to cell survival, cell death, and the regulation of function and/or disease. Studies also show NF-κB can influence mitochondrial function from outside the mitochondria. Therefore, it is essential to understand the complexity of these roles both inside and out of this organelle. In this review, an attempt is made to understand how NF-κB activity contributes to overall mitochondrial function – both inside and out. The discussion at times is speculative and perhaps even provocative to some, since NF-κB does not yet have defined mitochondrial targeting sequences for some nuclear-encoded mitochondrial genes and mechanisms of mitochondrial import for NF-κB are not yet entirely understood. Also, the data associated with the mitochondrial localization of proteins must be yet further proved with additional experiments.

## Introduction

Nuclear factor kappa B (NF-κB) is an ancient protein transcription factor (Salminen et al., 2008) and considered a regulator of innate immunity (Baltimore, 2009). The NF-κB signaling pathway links pathogenic signals and cellular danger signals thus organizing cellular resistance to invading pathogens. In fact, a plethora of studies have shown NF-κB is a network hub responsible for complex biological signaling (Albensi and Mattson, 2000; Kaltschmidt and Kaltschmidt, 2009; Karin, 2009). To this end, NF-κB has been hypothesized to be a master regulator of evolutionarily conserved biochemical cascades (Mattson et al., 2000). Other factors are also translocated into the mitochondria and are involved in modulating expression (Barshad et al., 2018a), but are not the focus of this review. The purpose of this review is to attempt to understand how NF-κB activity contributes to mitochondrial function. It is assumed the reader already has an understanding of basic mitochondrial biology. In the case of further study, the reader is referred to many excellent studies and reviews on mitochondrial structure and function (Hall, 1979; Fox, 1982; Roger and Silberman, 2002; Henze and Martin, 2003; Conradt, 2006; Ettema, 2016; Wang and Youle, 2016; Barshad et al., 2018b).

## NF-κB Activation

**FIGURE 1:**
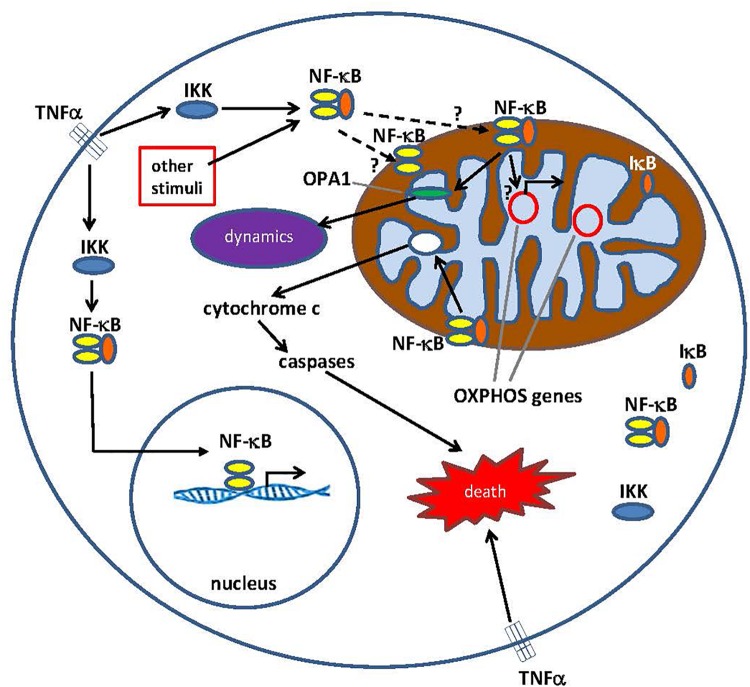
Pathways for Nuclear factor κ B (NF-κB) signaling in the cytoplasm and the mitochondrion. The NF-κB tri-subunt complex (e.g., p65, p50, IκB – one possible combination) exists in an inactive state in the cytoplasm. NF-κB activation is initiated when molecules such as TNFα bind to TNF receptors (different types exist). Other external or internal stimuli can also activate NF-κB. A complicated signal transduction process then begins once TNF receptors are activated; IκB kinase (IKK) is ultimately triggered and leads to the phosphorylation of IκB, which results in IκB ubiquitination and degradation. Once IκB is degraded, the remaining NF-κB dimer (e.g., p65/p50 or p50/p50 subunit combinations are possible) translocates to the nucleus, where it binds to a DNA consensus sequence of target genes. By processes not well understood, the NF-κB complex or NF-κB subunits can also migrate into the mitochondrion, where evidence suggests it/they occupies the intermembrane space. Once inside the mitochondria, NF-κB is thought to interact with OXPHOS genes (mitochondrial mtDNA) that leads to the expression of proteins involved in various functions, including mitochondrial dynamics and COX III regulation (component of Complex IV). Evidence also suggests, NF-κB can function as a switch in the mitochondria and control the balance between the utilization of cytoplasmic glycolysis and mitochondrial respiration in normal cells and in cancer. Finally, data also point to intrinsic apoptotic pathway stimulation, where NF-κB activation in the mitochondria leads to cytochrome c release, thus triggering caspase cascades and programed cell death.

Nuclear factor kappa B subunits, comprising the NF-κB complex, are expressed in both neurons and glia. The NF-κB complex exists in an inactive state in the cytoplasm (Ghosh et al., 1998; Aggarwal et al., 2004; Hayden and Ghosh, 2004) where the activation of NF-κB has been well described (Li and Karin, 2000; Baud and Jacque, 2008; Israel, 2010). When stimulated by molecules such as TNFα, or other cell stressors, TNFα binds to TNF receptors (Figure 1). This binding, via several intermediate steps, leads to an interaction with the IκB kinase (IKK) complex, which then leads to the phosphorylation of IκB, and subsequently results in IκB ubiquitination and degradation. Once degraded, the remaining NF-κB dimer (e.g., p65/p50 subunits) translocates to the nucleus, where it binds to the DNA consensus sequence of various target genes. The selectivity of the NF-κB response is based on several factors (Sen and Smale, 2010) including dimer composition, timing, and cell type. NF-κB’s influence on cell survival is also complex and can be neuroprotective or proinflammatory, depending on cell type, developmental stage, and pathological state (Qin et al., 2007).

Organizationally, NF-κB is a Rel family transcription factor and is associated with five genes, NF-κB1, NF-κB2, RELA, RELB, and REL (Chen and Greene, 2004); these genes encode several proteins, NF-κB1, NF-κB2, RelA, RelB, and c-Rel, respectively, where two of these proteins are large precursor proteins known as p105 and p100 that undergo proteolysis to become p50 and p52, respectively. These proteins contain REL-homology domains (RHD) at their amino-terminal region; the RHD region is composed of 2 separate, but adjoining domains. The sequence most distant from the carboxy-terminal region allows the protein to bind to DNA. A more interior sequence allows the Rel family proteins to dimerize (homo- or heterodimers) for the suppression of expression via the binding of their corresponding family of inhibitors, the IκB proteins (Chen and Greene, 2004). The latter sequence includes the nuclear-localization sequence (NLS) that becomes unmasked when IκB is unbound by degradation. The NLS has the job of guiding or tagging active proteins for import into the cell nucleus (Chen and Greene, 2004; Karin et al., 2004; Barger et al., 2005).

Three of these proteins (RelA, RelB, and c-REL) also encode a transactivation domain (TADs) in their carboxy-terminal region. The TADs allow these proteins to interact with the basal transcription apparatus, known as the TATA binding protein (TBP), Transcription Factor IIB, as well as the p300 and cAMP response element (CREB) binding protein (CBP) transcriptional co-activators (Chen and Greene, 2004). Only these three proteins are able to induce transcription of their DNA-coding regions while the other proteins, the p50 and p52 homodimers, are able to occupy the DNA binding sites without initiating transcription. Given this, the later 2 homodimers proteins of p50 and p52 act as transcriptional repressors (Chen and Greene, 2004).

The p105 and p100 homodimers occupy DNA binding sites thus blocking transcription via transcription factors that do possess TADs (Barger et al., 2005). A third form of transcriptional repression is due to IκB proteins. These proteins have several ankyrin repeats as their core domain and function by binding to the RHD that mask the NLS (Karin et al., 2004). Without an active NLS, the NF-κB proteins are restricted to the cytoplasm and are unable to migrate into the nucleus and so transcription is blocked.

## NF-κB Is Found in the Mitochondria

In 2001, a study by Bottero et al. (2001) found IκBα and the NF-κB p65 subunit in subcellular fractions and purified mitochondria from Jurkat cells. Jurkat cells are an immortalized cell line of human T lymphocyte cells that are used to study leukemia. In Bottero’s study, it was determined that IκBα and NF-κB p65 were localized in the mitochondrial intermembrane space. The mitochondrial intermembrane space is the space that exists between the inner mitochondrial membrane (IMM) and the outer mitochondrial membrane (OMM).

Subsequently, Cogswell et al. (2003) also showed that NF-κB subunits, p50 and p65, and IκBα, were found in the mitochondria. To determine this, several methods were used to provide evidence, including electron microscopy of sections of U937 cells. U937 cells were first isolated from the lymphoma of a middle-aged male patient to study the behavior and differentiation of monocytes. Here Cogswell et al. (2003) was able to visualize NF-κB p50 and p65 subunits and IκBα in the inner matrix of the mitochondria. Rat liver cells were also examined in this study and the p50 subunit and IκBα subunit were also identified. Additionally, U937 cells were stimulated for 1 h with TNFα, a known trigger of the NF-κB signaling pathway. In this experiment, Western blot analyses in mitochondrial and cytoplasmic fractions found that TNFα treatment caused a loss of IκBα in both mitochondrial and cytoplasmic compartments by 30 min following treatment suggesting that IκBα was degraded. EMSA analysis, an *in vitro* assay that detects NF-κB activation and non-specific binding to DNA sequences, was also conducted on protein taken from nuclear extracts from mitochondria isolated from U937 cells stimulated with TNFα. Here they determined TNFα signaling led to increased DNA binding activity of NF-κB p50, in protein taken from the mitochondria.

Other studies have also detected NF-κB in the mitochondria. These include studies (Guseva et al., 2004; Zamora et al., 2004) in human fibroblast HT1080 cell lines, human prostate LNCaP and PC3 cell lines, and HeLa cells. In LNCaP cells, mitochondria NF-κB p50 and p65 subunits were found bound to mitochondrial DNA (mtDNA). Taken together, these studies show evidence for NF-κB signaling in the mitochondria and that NF-κB regulates mitochondrial mRNA expression (see NF-κB and mitochondrial gene expression section below).

## NF-κB Controls Mitochondrial Dynamics

There are several proteins involved in the dynamics (fission and fusion) and morphology of the mitochondria (Karbowski and Youle, 2003; Olichon et al., 2006; Brooks and Dong, 2007; Song et al., 2008; Autret and Martin, 2010; Silva et al., 2013; Sinha and Manoj, 2019). One of these is the optic atrophy 1 protein (OPA1) (Olichon et al., 2006; Garcia et al., 2018; Lee and Yoon, 2018). Studies have suggested that OPA1 is a regulator of mitochondrial inner membrane fusion and also mitochondrial cristae remodeling (Cipolat et al., 2006). Recently Laforge et al. (2016) showed that the absence of IKKα had an impact on OPA1 expression in the mitochondria and on mitochondrial morphology.

Surprisingly, in a recent study by Nan et al. (2017), TNFα receptor 2 (TNFR2) stimulation was found to promote mitochondrial fusion via the NF-κB-dependent activation of OPA1 expression in cardiac myocytes. Importantly, TNFR2 activation in this study protected cardiac myocytes against stress by upregulating OPA1 expression. By administering low concentrations of exogenous TNFα (0.5 ng/mL) before ischemia-reperfusion appeared to enhance cell survival, whereas higher concentrations (10–20 ng/mL) led to toxic effects in cells.

## NF-κB and Apoptosis in the Mitochondria

Mitochondria’s role in programed cell death, or apoptosis has been known for quite some time (Green and Reed, 1998; Wang and Chen, 2015). The most important role for the mitochondria is the generation of ATP; however, the second most important function for the mitochondria is probably in controlling cell death. How does the mitochondrion do this? If the mitochondrion fails at triggering cell death, cancer is often the consequence. So, in order to regulate cell death, mitochondria integrate signals from a variety of sources, which are known as intrinsic pathways of apoptosis. Components of NF-κB activity appear to be one of these signals, although TNFα, an activator of NF-κB, is part of an extrinsic pathway of apoptosis. Extrinsic pathways (death receptor mediated) are initiated outside of the cell, whereas intrinsic pathways of apoptosis are mediated and triggered in the mitochondria.

In a recent study by Pazarentzos et al. (2014), IκBα was found to exert pro-apoptotic activity as it inhibited the anti-apoptotic NF-κB. In most cells, the activation of NF-κB leads to downstream target gene expression that triggers cell death resistance (Luo et al., 2005). In this study, it was shown that a novel apoptosis function was due to IκBα, the subunit that inhibits NF-κB’s activation. Pazarentzos et al. (2014) found that IκBα localizes to the OMM where it interacts with a voltage dependent anion channel (VDAC) and mitochondrial hexokinase II (HKII) to stabilize this complex and prevent Bax-mediated cytochrome c release for apoptosis. Bax is a member of the Bcl-2 family of proteins, which have been shown to be regulators of programed cell death (Karbowski et al., 2006).

Other studies have also hinted at NF-κB’s role in more directly regulating apoptosis in the mitochondrion. In a study by Liu et al. (2004), inhibition of NF-κB alone in macrophages resulted in the release of cytochrome c. Recall that cytochrome c is responsible for shuttling electrons from Complex III to Complex IV and that the release of cytochrome c into the cytoplasm, an activator of caspases, is a key step in triggering apoptosis.

## NF-κB and Mitochondrial Respiration

Nuclear factor kappa B has been shown in many studies to promote tumorigenesis. How this occurs was not exactly clear. In a groundbreaking study by Mauro et al. (2011), NF-κB was found to upregulate mitochondrial respiration in colon carcinoma cells. Here they established that this function of NF-κB suppresses the Warburg effect. Recall that the Warburg effect (Vander Heiden et al., 2009) describes the observation that cancer cells tend to favor metabolism by glycolysis rather than by the more efficient oxidative phosphorylation pathway. So in this study the authors determined that NF-κB organizes networks of energy metabolism by controlling the balance between glycolysis utilization and mitochondrial respiration. Interestingly, they found a role for NF-κB in metabolic adaptation in normal cells and in cancer. Their results further suggested that suppressing mitochondrial metabolism in established cancer cells by inhibition of NF-κB and metformin decreases tumorigenesis.

## NF-κB and Mitochondrial Gene Expression

Nuclear factor kappa B is a known regulator of gene expression – both negatively and positively (Mattson et al., 2000). However, how NF-κB regulates or influences nuclear-encoded mitochondrial gene expression is less understood. Human mtDNA possess 37 genes that encode for 13 polypeptides. It has been shown that mtDNA genes code for many of the subunits of all 5 complexes of the electron transport chain (ETC), 2 rRNAs, and 22 tRNAs. Albeit, most ETC subunits are coded by nuclear DNA, which could be influenced by NF-κB activity (Calvo et al., 2016).

For example, it has been claimed (Cogswell et al., 2003) that the NF-κB pathway can negatively regulate mitochondrial gene expression associated with the COX III subunit. The COX III subunit is encoded by mtDNA and is a component of Complex IV in the mitochondrial ETC. It functions as a catalytic subunit in Complex IV, which is the complex associated with mitochondrial oxygen consumption. In a study by Cogswell et al. (2003), modulation of NF-κB activation resulted in the loss of expression of both COX III and cytochrome b mRNA. Other studies support a role for NF-κB regulating additional mitochondrial genes, such as COX I, and Cytb (Psarra and Sekeris, 2008, 2009; Barshad et al., 2018b). Additionally, NF-κB p65 subunit reduced levels of mtDNA-encoded CytB mRNA, possibly by binding to the D-loop in human cells in the absence of p53 (Johnson et al., 2011). Overall, these results suggest that NF-κB signaling can influence the enzymatic activity of respiratory ETC complexes.

## NF-κB Mediates Aβ-Induced Dysfunction in the Mitochondria

Alzheimer’s disease (AD) is associated with the build-up of Aβ plaques and/or the appearance of neurofibrillary tangles (NFTs) in certain brain regions (Duyckaerts et al., 2009). However, controversy exists around whether Aβ is a causative agent of AD or if Aβ is simply correlated with aging. Accumulating evidence (Aliev et al., 2009; Correia et al., 2012; Cadonic et al., 2016; Cardoso et al., 2017; Djordjevic et al., 2017) also now points to changes in brain metabolism driven by mitochondrial dysfunction as a process central to many age-related neurodegenerative disorders including AD. Adding to this evidence, there are also impairments in enzymatic activity of the protein complexes of the ETC and alterations in antioxidant enzymatic activity (Kolosova et al., 2017) in AD. In particular, Complex IV activity has been shown to be negatively affected in AD (Mutisya et al., 1994).

In a recent study by Shi et al. (2014), it was found that Aβ impaired mitochondrial function via NF-κB signaling. Moreover, Shi et al. (2014) showed here that Aβ decreased the expression of the COX III subunit via a NF-κB pathway. Importantly, to eliminate the possibility that IκBα was phosphorylated by Aβ in the cytoplasm (and then transported into the mitochondria), isolated mitochondria were incubated with Aβ in the presence (or absence) of a NF-κB blocker, namely BAY11-7082. Here they found Aβ induced phosphorylation and degradation of IκBα in isolated mitochondria.

These findings also have important implications for AD treatment as demonstrated by recent studies by Snow et al. (2018) and as shown in other related studies (Djordjevic et al., 2017; Adlimoghaddam et al., 2019) that suggest targeting NF-κB signaling in the mitochondria may have therapeutic value. For example, in Snow et al.’s study, creatine – a known modulator of mitochondrial function (Tarnopolsky and Beal, 2001), was shown to increase and positively alter protein levels of CaMKII, PSD-95, and Complex 1 subunits in creatine fed mice, whereas the NF-κB inhibitory IκB subunit was decreased. For additional reading on creatine’s potential therapeutic effect on mitochondrial function and in mitochondrial disorders or other neurological disorders see studies and reviews by Matthews et al. (1998), Klivenyi et al. (1999), Tarnopolsky and Beal (2001), Hersch et al. (2006), Rodriguez et al. (2007) and Beal (2011).

## NF-κB’s Role in Inflammation and Mitochondrial Metabolism

Increasing data (Lamas et al., 2003; Mauro et al., 2011; Moretti et al., 2012) are suggesting that NF-κB signaling, which is a mediator of inflammatory processes, is also functioning as a regulator and integrator with energy metabolism. In a recent study by Zhong et al. (2016), NF-κB was shown to restrict inflammasome activation via elimination of damaged mitochondria. Surprisingly, NF-κB appeared to both prime the NLRP-3 inflammasome for activation *and* also prevented excessive inflammation and restrained NLRP-3 inflammasome activation; although the mechanism for restraint was poorly defined. Here it was speculated that in addition to NF-κB being an activator of inflammatory genes, it also functioned in this study by limiting NLRP3 inflammasome activation and IL-1β production. Moreover, it was found that p62 induction was responsible for the inflammasome inhibitory activity by NF-κB. It appears that NF-κB can restrain its own inflammation in macrophages by promoting p62 mediated removal of damaged mitochondria (mitophagy) after macrophages interact with different NLRP3 inflammasome activators.

## Conclusion

Over 10 years ago, NF-κB was detected in the mitochondria. Surprisingly, for such an important transcription factor, little progress has been made in uncovering specific roles for NF-κB affecting the mitochondrion. Some studies, as described above, do provide evidence for NF-κB in mitochondrial dynamics, apoptosis, respiratory control, gene expression, and mechanisms of disease (Figure 1). However, duplication of these results and overall validation is still necessary by other laboratories. Some additional insight may be gleaned from the fact that other transcription factors having effects on nuclear genes, such as AP-1, p53, CREB, c-Myc, Wnt13, Dok-4, HMGA1, and c-Src have also been detected in the mitochondria (Psarra and Sekeris, 2008). Interestingly, binding sites in the mitochondrial genome (homologous to their binding sites in the nuclear DNA) for some of these factors have been determined (Psarra and Sekeris, 2008) where roles for mitochondrial transcription and apoptosis are suspected and show some overall patterns of activity. For example, an argument can be made that some of these factors (NF-κB, CREB, and AP-1) bind to mitochondrial genomes and mostly attenuate mitochondrial gene expression (Blumberg et al., 2014), while having stimulatory effects on nuclear gene transcription. However, clearly more work is needed to not only find precise roles of activity, but also to determine if overall patterns of activity truly exist.

After surveying this literature, it also becomes apparent that NF-κB’s role in the regulation of mitochondrial respiration has profound implications and demonstrates a level of complexity not previously appreciated. For instance, Mauro et al. (2011) data establish a role for NF-κB in metabolic adaptation in normal cells and in cancer, and also suggest consequences for other disease states such as AD. Furthermore, given that NF-κB can restrain its own inflammation as shown by Zhong et al. (2016), not only is surprising, but further exemplifies the complexity of NF-κB signaling in mitochondrial function.

In this review, studies were surveyed on NF-κB’s role in mitochondrial function, and it appears that research in this area is increasing. Complicating the results though is the observation that multiple factors are playing similar roles in mitochondrial function and so detailed studies specific to each factor are necessary. In conclusion, we can re-ask the question – *What is NF-*κ*B doing in and to the mitochondrion?* The immediate and abbreviated reply would be – *a lot*!

## Author Contributions

The author created the topic and wrote the manuscript.

## Conflict of Interest Statement

The author declares that the research was conducted in the absence of any commercial or financial relationships that could be construed as a potential conflict of interest.
